# The influence of maternal smoking habits on the risk of subsequent stillbirth: is there a causal relation?

**DOI:** 10.1111/j.1471-0528.2007.01340.x

**Published:** 2007-06-01

**Authors:** L Högberg, S Cnattingius

**Affiliations:** Department of Medical Epidemiology and Biostatistics, Karolinska Institutet Stockholm, Sweden

**Keywords:** Pregnancy, preucation, risk, smoking, still birth

## Abstract

**Objective:**

Maternal smoking has previously been associated with risk of stillbirth. If women who quit smoking reduce their risk of stillbirth, the hypothesis of a causal association would be supported.

**Design:**

Prospective cohort study.

**Setting:**

Nationwide study in Sweden.

**Population:**

All primiparous women who delivered their first and second consecutive single births between 1983 and 2001, giving a total number of 526 691 women.

**Method:**

A population-based Swedish study with data from the Medical Birth Registry, the Immigration Registry and the Education Registry. Logistic regression analyses were used to estimate odds ratios, using 95% confidence intervals.

**Main outcome measure:**

Stillbirth in the second pregnancy.

**Results:**

Compared with nonsmokers in both pregnancies, women who smoked during the first pregnancy but not during the second do not have an increased risk of stillbirth (OR 1.02; 95% CI 0.79–1.30), while corresponding risk among women who smoked during both pregnancies was 1.35 (95% CI 1.15–1.58).

**Conclusion:**

The result supports that maternal smoking during pregnancy is causally associated with stillbirth risk. Smoking is a preventable cause of stillbirth, and smoking interventions is an important issue in antenatal care.

## Introduction

Stillbirth is a relatively rare adverse pregnancy outcome in western countries such as Sweden (3.4 per 1000 live births^1^), but when it happens, it causes great emotional distress for the parents. A lot of effort is devoted to investigate risk factors and causes of stillbirth.

Smoking has repeatedly been associated with risk of stillbirth, and risks generally increase with the amount smoked. The hypothesis of a causal relation would be supported if an altered exposure to cigarette smoke, for example smoking cessation, could be shown to reduce the risk of stillbirth. In a Danish cohort study, Wisborg *et al.*^2^ found that risk of stillbirth to mothers who stopped smoking during the first trimester was comparable to the risk among women who were nonsmokers during the entire pregnancy. In contrast, a Canadian study found that stillbirth risk for women who had quit smoking by 16 weeks was higher than corresponding risk among nonsmokers and similar to that for women who smoked throughout pregnancy.^3^

In a nationwide Swedish register study, we included more than 500 000 women with two successive single births. Since information about maternal smoking was available, this allowed us to study the effects of changed smoking habits in relation to the risk of stillbirth in second pregnancy.

## Methods

### Study design

The Swedish Medical Birth Register includes prospectively collected information on virtually all births in Sweden. Maternal characteristics are obtained by midwives in a standardised manner at the first visit to antenatal care, which occurs before the 15th week of gestation in more than 95% of the pregnancies.^4^ Information about smoking habits is also recorded at this occasion in standardised antenatal records with check boxes and is categorised into three groups: nonsmoking (nonsmoking daily), moderate smoking (one to nine cigarettes per day) or heavy smoking (at least ten cigarettes per day). Information on smoking habits has been recorded since 1983. Using each mother’s unique national registration number, it is possible to link information on successive births in the birth register. In this study, we included women who had their first and second successive single births between 1983 and 2001, a total number of 526 691 women.

Information about the mother’s country of birth and the education level were obtained by linking the Medical Birth Register to the Immigration and Education Registers, using the mothers’ unique national registration numbers. Country of birth was grouped as Nordic (Sweden, Finland, Norway, Denmark and Iceland) or non-Nordic. Education was categorised as ≤9, 10–11, 12, 13–14 and ≥15 years of formal education, as completed by the end of year 2001. The interpregnancy interval was calculated as the number of completed months between the birth of the first child and estimated date of conception of the second child and categorised as <1, 1 to <3 and ≥3 years. To determine gestational age, early second-trimester ultrasonographic examinations were used when available, otherwise the last menstrual period was used. Early second-trimester ultrasound screening was successively introduced in Sweden in the 1980s. All pregnant women in Sweden have been offered this examination since 1990, and 95% accepted the procedure.^5^ Stillbirth was defined as fetal death after at least 28 completed weeks of gestation. The study was approved by the research ethics committee at Karolinska Institutet, Stockholm, Sweden.

### Statistical methods

We used logistic regression analyses to evaluate associations between maternal smoking habits in the two pregnancies and risk of stillbirth. Both univariable and multivariable analyses were performed and are presented as crude and adjusted odds ratios, with 95% confidence intervals. The multivariable analyses were adjusted for effects of maternal age, educational level, cohabitation with the infant’s father, the mother’s country of birth, year of delivery, interpregnancy interval and stillbirth in the first pregnancy. All analyses were performed using the Statistical Analysis Software version 9.1 (SAS Institute, Inc., Cary, NC, USA).

## Results

Smoking habits during first and second pregnancies in relation to stillbirth risk in second pregnancy is presented in Table 1. In the crude analyses, risk of stillbirth in second pregnancy increases with the amount smoked in both first and second pregnancies. However, the majority (64.6% of moderate smokers and 81.4% of heavy smokers) of women who smoked during the first pregnancy also smoked while pregnant with the second child. When we adjusted for smoking in second pregnancy and other factors, smoking in first pregnancy was no longer associated with stillbirth risk in second pregnancy. In contrast, among smokers in the second pregnancy, corresponding adjusted risk was increased in heavy (≥10 cigarettes/day) smokers (OR 1.45; 95% CI 1.16–1.81) but not in moderate (one to nine cigarettes/day) smokers (OR 1.10; 95% CI 0.89–1.35). Thus, the smoking-related risk of stillbirth in second pregnancy was confined to smoking in second pregnancy.

**Table 1:** Smoking during first and second pregnancy in relation to stillbirth risk in second pregnancy

	Number of deliveries (*n* = 526 691)	Stillbirth in second pregnancy			
		
		*n*	Rate/1000	Odds ratio (95% CI)	
				
				Crude	Adjusted*
**Smoking in first pregnancy**					
Nonsmoker**	389 362	992	2.5	1.00	1.00
1–9 cig./day	70 740	216	3.1	1.20 (1.04–1.39)	1.13 (0.92–1.39)
≥10 cig./day	35 348	133	3.8	1.48 (1.23–1.77)	1.14 (0.87–1.51)
Data missing	31 241	79	2.5		
**Smoking in second pregnancy**					
Nonsmoker**	407 710	985	2.4	1.00	1.00
1–9 cig./day	56 929	161	2.8	1.17 (0.99–1.38)	1.10 (0.89–1.35)
≥10 cig./day	31 149	120	3.9	1.60 (1.32–1.93)	1.45 (1.16–1.81)
Data missing	30 903	154	5.0		

Cig., cigarettes.

* Adjusted for the effects of maternal age, education, cohabiting with infant’s father, mother’s country of birth, year of second delivery, interpregnancy interval and stillbirth in first pregnancy. Odds ratios related to smoking in first pregnancy are adjusted for smoking in second pregnancy, and odds ratios related to smoking in second pregnancy are adjusted for smoking in first pregnancy.

** Reference group.

Risks of stillbirth in the second pregnancy in relation to maternal characteristics are presented in Table 2. Women with a previous stillborn baby faced a more than doubled increase in risk of stillbirth in the second pregnancy (OR 2.42; 95% CI 1.32–4.41). Maternal smoking during both first and second pregnancies involves a significantly increased risk (OR 1.35; 95% CI 1.15–1.58), while women who only smoked during the first pregnancy did not have an increased risk of stillbirth in the second pregnancy (OR 1.02; 95% CI 0.79–1.30). No effect on risk was seen in women who stated that they were smokers only in the second pregnancy (OR 0.84; 95% CI 0.55–1.26). The risk of second pregnancy stillbirth was also increased among women with a high maternal age (≥35 years), a non-Nordic country of birth and long interpregnancy intervals (≥3 years). Low maternal age (≤19 years) was associated with a relatively high stillbirth rate in second pregnancy (4.7 per 1000). However, after adjustment for smoking and other variables, low maternal age did not increase the risk of stillbirth. The risk of stillbirth was reduced among women with high maternal education.

**Table 2:** Characteristics of women who delivered two successive singleton infants between 1983 and 2001 in Sweden and associations with the risk of stillbirth

Characteristic	Number of second deliveries (*n* = 526 691)	Second delivery stillbirth (*n* = 1420)		Odds ratio (95% CI)
			
		*n*	Rate/1000	Adjusted*
**Stillbirth at first delivery**				
No**	524 328	1402	2.7	1.00
Yes	2363	18	7.6	2.42 (1.32–4.41)
**Smoking status**				
Nonsmoker in both pregnancies**	356 980	863	2.4	1.00
Nonsmoker in first pregnancy and smoker in second pregnancy	12 039	26	2.2	0.84 (0.55–1.26)
Smoker in both pregnancies	69 388	236	3.4	1.35 (1.15–1.58)
Smoker in first pregnancy and nonsmoker in second pregnancy	29 471	79	2.7	1.02 (0.79–1.30)
Data missing	58 813	216	3.7	
**Maternal age at second delivery (years)**				
≤19	2971	14	4.7	1.38 (0.70–2.70)
20–24	92 398	216	2.3	0.88 (0.74–1.06)
25–29**	220 412	558	2.5	1.00
30–34	159 569	430	2.7	1.02 (0.88–1.18)
≥35	51 340	202	3.9	1.39 (1.15–1.69)
Data missing	1	0	0	
**Education (years)**				
≤9	51 209	179	3.5	1.10 (0.90–1.34)
10–11**	183 599	517	2.8	1.00
12	99 434	249	2.5	0.87 (0.73–1.03)
13–14	96 680	243	2.5	0.86 (0.72–1.03)
≥15	90 710	214	2.4	0.82 (0.68–0.99)
Data missing	5059			
**Living with the baby’s father**				
Yes**	478 690	1221	2.6	1.00
No	13 547	47	3.5	1.09 (0.79–1.51)
Data missing	34 454	152	4.4	
**Mother’s country of birth**				
Nordic country**	482 706	1222	2.5	1.00
Other country	42 929	161	3.8	1.45 (1.19–1.76)
Data missing	1056	37	35.0	
**Interpregnancy interval (years)**				
<1	101 656	254	2.5	1.01 (0.86–1.19)
1 ≤ 3**	307 813	748	2.4	1.00
≥3	116 287	406	3.5	1.31 (1.14–1.51)
Data missing	935	12	12.8	
**Year of second delivery**				
1983–89	124 455	325	2.6	1.03 (0.87–1.22)
1990–93**	151 975	383	2.5	1.00
1994–97	134 552	363	2.7	0.97 (0.83–1.15)
1998–2001	115 709	349	3.0	1.09 (0.92–1.29)

* Adjusted for the effects of maternal age, education, cohabiting with infant’s father, mother’s country of birth, interpregnancy interval, stillbirth in the first pregnancy and year of second delivery.

** Reference group.

Table 3 shows more detailed information of maternal smoking habits in first and second pregnancies in relation to risk of second pregnancy stillbirth. Risk of stillbirth in the second pregnancy was similar among women who were nonsmokers in both pregnancies and among women who were former (moderate or heavy) smokers but did not smoke during the second pregnancy. Compared with nonsmokers in both pregnancies, women who were smokers in both pregnancies generally experienced increased risks of stillbirth in the second pregnancy. No effects on risks were seen for women who stated that they were nonsmokers during the first pregnancy but smokers during the second pregnancy.

**Table 3:** Stillbirth rates in second pregnancy in relation to smoking habits in first and second pregnancy

Smoking in first pregnancy/smoking in second pregnancy	Stillbirth in second pregnancy		Odds ratio	
		
	*n*	Rate/1000	Crude	Adjusted*
None/none**	863	2.4	1.00	1.00
None/1–9 cigarettes per day	20	2.0	0.85 (0.54–1.32)	0.82 (0.52–1.30)
None/≥10 cigarettes per day	6	2.6	1.09 (0.49–2.45)	0.92 (0.38–2.22)
1–9 cigarettes per day/none	66	2.8	1.17 (0.91–1.50)	1.11 (0.85–1.44)
1–9 cigarettes per day/1–9 cigarettes per day	88	2.8	1.15 (0.92–1.43)	1.16 (0.92–1.46)
1–9 cigarettes per day/≥10 cigarettes per day	43	4.0	1.64 (1.21–2.23)	1.56 (1.13–2.16)
≥10 cigarettes per day/none	13	2.1	0.88 (0.51–1.51)	0.67 (0.36–1.26)
≥10 cigarettes per day/1–9 cigarettes per day	40	3.6	1.50 (1.09–2.06)	1.41 (1.01–1.96)
≥10 cigarettes per day/≥10 cigarettes per day	65	4.1	1.70 (1.32–2.19)	1.55 (1.17–2.04)

* Adjusted for the effects of maternal age, education, cohabiting with infant’s father, mother’s country of birth, interpregnancy interval, stillbirth in the first pregnancy and year of (second) delivery.

** Reference group.

## Discussion

We found that women who quit smoking from first to second pregnancy reduced their risk of stillbirth to the same level as nonsmokers in both pregnancies. These results indicate a causal relation between smoking and stillbirth. However, the finding that women who were smokers in second but not in first pregnancy had similar risk of stillbirth in the second pregnancy as nonsmokers in both pregnancies complicates the interpretation of the study results. If the smoking-related risk of stillbirth is caused by a direct toxic effect of smoking exposure during pregnancy, women who smoked in the second pregnancy should, irrespective of previous smoking habits, have an increased risk of stillbirth.

Smoking is causally associated with fetal growth restriction and probably also with placental abruption, which are two main causes of stillbirth.^6^ The association between smoking and stillbirth risk has previously been entirely explained through increased prevalence of placental complications and fetal growth restriction.^7^ Thus, there is a plausible biologic pathway by which smoking influences stillbirth risk. Furthermore, studies of smoking and stillbirth risk show consistent results, and dose–response relationships have generally been obtained.^8^ These findings lend support to the hypothesis that smoking during pregnancy may cause stillbirth.

The finding that women who only smoked in second pregnancy were not at increased risk of stillbirth may question that the association between smoking and stillbirth is mediated by a direct toxic effect during pregnancy. However, this finding may also have other causes. First, smoking was measured in early pregnancy, and 20–40% of women quit smoking during pregnancy.^8^ Second, the success rate of smoking cessation during pregnancy is substantially influenced by factors reflecting level of addiction, including age at onset of smoking and possibly other factors not accounted for in the analyses.^8^ Women who reported that they abstained from smoking early in the first pregnancy but not early in the second pregnancy are probably less addicted to cigarette smoking than persistent smokers. These women may therefore be more likely to quit smoking later during pregnancy, and smoking cessation in early pregnancy may reduce stillbirth risk.^2^ Third, women who take up smoking in second pregnancy may also differ from persistent smokers with respect to other unmeasured risk factors for stillbirth, including body mass index (BMI), weight change between pregnancies and obstetric history.^9^,^10^ Fourth, the group of women who only smoked in the second pregnancy was substantially smaller than the other groups. Thus, the results may, despite the large study population, have been a chance finding because of low statistical power.

This study included virtually all women with first and second consecutive single births in Sweden during the study period. The population-based design favours generalisability of findings at least across Sweden. Information on smoking habits among the more than 500 000 women was collected prospectively in early pregnancy, which precludes recall bias. We adjusted for maternal characteristics previously associated with stillbirth, such as a history of stillbirth, maternal age, maternal education and country of birth.^11^–^13^

We lacked information on other potential confounders, including use of alcohol or illicit drugs, exposure to passive smoking and maternal BMI.^14^–^17^ Information about smoking was only available in early pregnancy, and some women quit smoking later during pregnancy.^18^ By measuring smoking in early pregnancy, we may have underestimated the smoking-related risk of stillbirth. The reliability of self-reported smoking habits can be questioned because of decreasing public tolerance towards smoking during pregnancy. This may be especially true for later years, and we therefore included year of second delivery as a covariate in the analyses. Self-reported smoking information during pregnancy has an acceptable high validity,^19^ but women may underreport their smoking habits, either denying the habit or underestimating the number of cigarettes smoked daily.^20^,^21^ However, in a large population-based study, it would be hard to practically and cost-effectively use biochemical methods to verify smoking status.^22^,^23^

Although there has been a decreasing trend over the past 20 years, smoking is still common among pregnant women. In 1983, 31% of pregnant women in Sweden were daily smokers in early pregnancy, while corresponding figure in 2003 was 10% (results are available at www.socialstyrelsen.se/Publicerat/2005/8997/2005-125-14.htm). A decreasing number of smokers in society in general and among pregnant women in particular raises questions about why the rates of stillbirth have not decreased in a corresponding way. Perhaps, the answer can be found in a concurrent increase in maternal BMI and maternal age, which have been pointed out as risk factors for stillbirth.^6^,^9^,^12^,^14^ These factors will, provided that these increasing trends continue, probably play a larger role in the future.

The results from this study support the hypothesis that there is a causal relation between smoking and stillbirth risk. Heavy smokers are at more increased risk than moderate smokers, and women who manage to quit smoking between pregnancies show no increased risk of stillbirth in the second pregnancy. These results also support that the mechanism may involve a direct toxic effect of smoking exposure during pregnancy. Smoking interventions have been proven to increase smoking abstinence during pregnancy.^24^ As smoking is a preventable risk factor for stillbirth and other adverse pregnancy outcomes, smoking interventions should continue to have a high priority in the work of antenatal care centres.

## References

[1] The National Board of Health and Welfare Medical birth registration in 2003. www.socialstyrelsen.se/NR/rdonlyres/F11FF46F-7D50-4D78-AF54-0330B5E92239/3628/2005424.pdf.

[2] Wisborg K, Kesmodel U, Brink Henriksen T, Fródi Olsen S, Secher NJ (2001). Exposure to tobacco smoke in utero and the risk of stillbirth and death in the first year of life. Am J Epidemiol.

[3] Dodds L, King W, Fell D, Armson B, Allen A, Nimrod C (2006). Stillbirth risk factors according to timing of exposure. Ann Epidemiol.

[4] Lindmark G, Cnattingius S (1991). The scientific basis of antenatal care. Report from a state-of-the-art conference. Acta Obstet Gynecol Scand.

[5] Hogberg U, Larsson N (1997). Early dating by ultrasound and perinatal outcome. A cohort study. Acta Obstet Gynecol Scand.

[6] Cnattinius S, Stephansson O (2002). The epidemiology of stillbirth. Semin Perinatol.

[7] Raymond EG, Cnattingius S, Kiely JL (1994). Effects of maternal age, parity and smoking on the risk of stillbirth. Br J Obstet Gynaecol.

[8] Cnattingius S (2004). The epidemiology of smoking during pregnancy: smoking prevalence, maternal characteristics, and pregnancy outcomes. Nicotine Tob Res.

[9] Villamor E, Cnattingius S (2006). Interpregnancy weight change and risk of adverse pregnancy outcomes: a population based study. Lancet.

[10] Surkan PJ, Stephansson O, Dickman PW, Cnattingius S (2004). Previous preterm and small-for-gestational-age births and the subsequent risk of stillbirth. N Engl J Med.

[11] Robertson E, Malmström M, Johansson SE (2005). Do foreign-born women in Sweden have an increased risk of non-normal childbirth?. Acta Obstet Gynecol Scand.

[12] Astolfi P, De Pasquale A, Zonta L (2005). Late childbearing and its impact on adverse pregnancy outcome: stillbirth, preterm delivery and low birth weight. Rev Epidemiol Sante Publique.

[13] Olsen O, Madsen M (1999). Effects of maternal education on infant mortality and stillbirths in Denmark. Scand J Public Health.

[14] Brynhildsen J, Sydsjö A, Norinder E, Ekholm Selling K, Sydsjö G (2006). Trends in body mass index during early pregnancy in Swedish women 1978-2001. Public Health.

[15] Kesmodel U, Wisbort K, Olsen SF, Henriksen TB, Secher NJ (2002). Moderate alcohol intake during pregnancy and the risk of stillbirth and death in the first year of life. Am J Epidemiol.

[16] Ornoy A (2002). The effects of alcohol and illicit drugs on the human embryo and fetus. Isr J Psychiatry Relat Sci.

[17] Ahlborg G, Bodin L (1991). Tobacco smoke exposure and pregnancy outcome among working women. A prospective study at prenatal care centers in Orebro County, Sweden. Am J Epidemiol.

[18] The National Board of Health and Welfare. Medical birth registration in 2003 www.socialstyrelsen.se/NR/rdonlyres/C9098AD7-A8FE-4998-A0FF-F594FAE2A72A/4531/200512514.pdf.

[19] Lindqvist R, Lendahls L, Tollbom Ö, Åberg H, Håkansson A (2002). Smoking during pregnancy: comparison of self-reports and cotinine levels in 496 women. Acta Obstetricia et Gynecologica Scandinavica.

[20] Walsh RA, Redman S, Adamson L (1996). The accuracy of self-report of smoking status in pregnant women. Addict Behav.

[21] Britton GR, Brinthaupt J, Stehle JM, James GD (2004). Comparison of self-reported smoking and urinary cotinine levels in a rural pregnant population. J Obstet Gynecol Neonatal Nurs.

[22] Velicer WF, Diclemente CC, Rossi JS, Prochaska JO (1990). Relapse situations and self-efficacy: an integrative model. Addict Behav.

[23] Glasgow RE, Mullooly JP, Vogt TM, Stevens VJ, Lichtenstein E, Hollis JF (1993). Biochemical validation of smoking status: pros, cons, and data from four low-intensity intervention trials. Addict Behav.

[24] Pbert L, Ockene JK, Zapka J, Ma Y, Coins KV, Oncken C (2004). A community health center smoking-cessation intervention for pregnant and postpartum women. Am J Prev Med.

